# SIRT1 activator (SRT1720) improves the follicle reserve and prolongs the ovarian lifespan of diet-induced obesity in female mice via activating SIRT1 and suppressing mTOR signaling

**DOI:** 10.1186/s13048-014-0097-z

**Published:** 2014-10-21

**Authors:** Xiao-Ling Zhou, Jin-Jie Xu, Yan-Hong Ni, Xiao-Chun Chen, Hong-Xia Zhang, Xing-Mei Zhang, Wei-Juan Liu, Li-Li Luo, Yu-Cai Fu

**Affiliations:** Department of Gynaecology and Obstetrics of the First Affiliated Hospital, Shantou University Medical College, Shantou, Guangdong Province 515041 People’s Republic of China; Laboratory of Cell Senescence, Shantou University Medical College, 22 Xin Ling Rd, Jinping District, Shantou, Guangdong Province 515041 People’s Republic of China; Department of Gynaecology and Obstetrics of Huizhou Municipal Central Hospital, Huizhou, Guangdong Province 516001 People’s Republic of China

**Keywords:** SRT1720, Nicotinamide, Ovarian development, Obesity, Mice

## Abstract

**Background:**

The prevalence of obesity is increasing worldwide and significantly affects fertility and reproduction in both men and women. Our recent study has shown that excess body fat accelerates ovarian follicle development and follicle loss in rats. The aim of the present study is to explore the effect of SIRT1 activator SRT1720 on the reserve of ovarian follicle pool and ovarian lifespan of obese mice and the underlying mechanism associated with SIRT1 and mTOR signaling.

**Methods:**

Adult female Kunming mice (n = 36) were randomly divided into three groups: the normal control (NC) group (n = 8), the caloric restriction (CR) group (fed 70% food of the NC group, n = 8) and the high-fat diet (HF) group (fed a rodent chow containing 20% fat, n = 20). After 4 months, the HF mice were further randomly divided into three groups: the control high-fat diet (CHF, n = 8) group (treated every day with an intraperitoneal injection of vehicle), the SRT1720 (SRT, n = 6) group (treated every other day with an intraperitoneal injection of SRT1720 (50 mg/kg)), the SRT1720 and nicotinamide (NAM, n = 6) group (treated every other day with an intraperitoneal injection of SRT1720 (50 mg/kg) and every day with an intraperitoneal injection of nicotinamide (100 mg/kg)). After 6 weeks of treatment, ovaries were harvested for histological and Western blotting analyses.

**Results:**

The body weight, ovary weight and visceral fat in the SRT group were significantly lower than those in the CHF group at the end of treatment. Histological analysis showed that the SRT mice had significantly greater number and percentage of primordial follicles, but lower number and percentage of corpora lutea and atretic follicles than the CHF mice and NAM mice. Western blot analysis demonstrated that the levels of SIRT1, SIRT6, FOXO3a and NRF-1 protein expression significantly increased in the ovaries of SRT mice, whereas those of mTORC1, p-mTOR, p-p70S6K, NFκB and p53 decreased compared to the CHF and NAM mice.

**Conclusions:**

Our study suggests that SRT1720 may improve the follicle pool reserve in HF diet-induced obese female mice via activating SIRT1 signaling and suppressing mTOR signaling, thus extending the ovarian lifespan.

## Introduction

The prevalence of obesity has steadily increased over the past three decades all over the world, and newly released data has showed that nearly one-third of the world’s population is obese or overweight [1]. Significant evidence suggests that excess body fat is a major risk factor for non-insulin-dependent diabetes mellitus, cardiovascular diseases, cancers, gastrointestinal diseases, arthritis and metabolic disorders [2], as well as disruptions in reproduction [3]. Excess body fat (particularly abdominal obesity) is closely related to irregular menstrual cycles, reduced spontaneous conception and increased risk of miscarriage [4,5]. A recent study indicated that obesity negatively impacted oocyte and embryo quality [6]. In parallel to findings in human beings, diet-induced obese mouse studies have shown a wide range of negative reproductive phenotypes in addition to poor outcomes in the offspring from these mice [7-9]. Additionally, our previous study demonstrated that obesity accelerated ovarian follicle development and follicle loss in female rats [10]. Female fertility is determined by the size of the primordial follicle pool formed during fetal life and by the rate of depletion of the pool after birth [11]. In addition to reduced ovarian complement, early depletion of the follicular pool due to excess follicular activation and/or atresia can occur and results in infertility [12,13]. Childhood obesity also has a negative effect on reproduction, which may lead to early onset of puberty, menstrual irregularities during adolescence and polycystic ovary syndrome [14]. These studies shed light on the negative effects of obesity on the reproductive functions in females. However, how obesity affects the ovarian follicle development, and the underlying mechanisms remain elusive.

Anti-obesity management can improve cardiovascular and diabetes risk factors in overweight and obese individuals [15], as well as reproduction disease [16]. Resveratrol, a natural SIRT1 activator, can partly mimic effects of calorie restriction (CR) in mice and obese humans [17,18]. Resveratrol has anti-aging effect and also beneficial effects of cardiovascular and metabolic system [19,20]. Consistently, it prolongs the ovarian lifespan and protects against age-associated infertility in rodents [21,22]. However, resveratrol is not a specific activator of SIRT1, and it can also activate other signaling pathways [23]. SRT1720, a specific activator of SIRT1, is 1000 times more potent than resveratrol [24,25]. However, whether SRT1720 could affect ovarian follicle development and promote the follicle pool reserve through activating SIRT1 signaling is unknown. In the present study, we used a high-fat diet induced obese mouse model to characterize the effect of SRT1720 on ovarian follicle development in adult obese animals and to investigate the associated mechanism with SIRT1 and mTOR signaling.

## Materials and methods

### Materials

Primary and secondary antibodies applied in this study were introduced as follows: SIRT1, FOXO3a, NRF-1, mTOR, phospho-mTOR, phospho-p70S6 kinase α, NF-κB and p53 antibodies were obtained from Santa Cruz Biotechnology, USA; SIRT6 antibody was purchased from Abcam, UK; β-actin was obtained from Zhongshan Golden Bridge, Beijing, China; Secondary antibodies to mouse and rabbit IgG were purchased from Sigma, USA. SRT1720 was obtained from Selleck, USA, and nicotinamide was obtained from Sigma, USA. They were dissolved in double distilled water containing 10% ethanol and 40% polyethylene glycol 400 for intraperitoneal injection [25].

### Animals and regiments

Thirty six female Kunming mice (4 weeks old, 20 ± 2 g) were purchased from Shantou University Medical College Laboratory Animal Center. After 4 weeks of adaptation, mice were randomly divided into three diet groups: the normal control (NC) group (n = 8) fed *ad libitum* a standard rodent chow (4.84% fat,7.34% fiber, 20.11% protein, plus all necessary vitamins and minerals, GE =3.5 Kcal/g), the high-fat (HF) group (n = 20) fed *ad libitum* a high-fat chow purchased from Shanghai Laboratory Animal Center (20.0% fat, 21.0% protein, 43.5% carbohydrate, plus all necessary vitamins and minerals, GE =4.383 kcal/g), and the CR group (n = 8) fed 70% of the food intake from the NC group. We recorded daily food intake of the NC mice, and the food supply of the CR group was adjusted accordingly. After 4 months of high-fat diet treatment, the HF mice were further randomly divided into three groups: the control high-fat diet (CHF) group (n = 8) (treated every day with an intraperitoneal injection of vehicle), the SRT1720 (SRT) group (n = 6) (treated every other day with an intraperitoneal injection of SRT1720 (50 mg/kg)), the nicotinamide and SRT1720 (NAM) group (n = 6) (treated every other day with an intraperitoneal injection of SRT1720 (50 mg/kg) and every day with an intraperitoneal injection of nicotinamide (100 mg/kg) ). They were maintained on these treatments for 6 weeks.

All of the mice were housed 2 in steel cages in a room with an ambient temperature of 22°C ±2°C and a 12-hour light:12-hour dark cycle and had free access to tap water. All animal protocols were approved by the Institutional Animal Care and Use Committee of Shantou University Medical College.

### Estrous cycle analysis

Vaginal smears of all mice were taken daily between 9:00 and 10:00 A.M. Vaginal cells were collected via a sterile cotton swab moistened with normal saline, and then placed on a clean glass slide. Stages were analyzed under the microscope and assessed based on vaginal cytology [26]. A 4 to 5-day estrous cycle was determined to be a regular cycle, and a cycle duration of >5 days or <4 days was considered to be an irregular cycle [27].

### Preparation of ovarian sections

The mice were weighed every four weeks. After 24 weeks, mice were anaesthetized at the diestrus phase of the cycle with pentobarbital sodium at 40 mg/kg body weight, and sacrificed by cervical dislocation. Mouse perirenal fat was isolated and weighed and expressed as visceral fat index. Both ovaries of each mouse were removed and weighed. One ovary was stored at −80°C for Western blot analysis, and the other one was fixed in 4% paraformaldehyde at room temperature for 4 hours, flushed under running water for 3 hours, then dehydrated through a series of concentrations of ethanol, cleared in xylene and embedded in paraffin. Ovarian sections of 4 μm were prepared for hematoxylin and eosin (HE) staining.

### HE staining and follicle classification

The sections were deparaffinized in xylene, hydrated with decreasing alcohol concentrations, and stained with HE using standard protocols. Sections were mounted using Canada balsam and observed under a light microscope. Five representative sections from each ovary were selected for follicle counting, with each observed section separated by a distance of over 80 μm. Follicles were classified according to a previous study [28] as follows: primordial follicle (an oocyte surrounded by one layer of flattened granulosa cells), primary follicle (an oocyte surrounded by one layer of cuboidal granulosa cells), secondary follicle (two or three layers of cuboidal granulosa cells with no antral space), and antral follicle (more than four layers of granulosa cells with one or more independent antral spaces). In some cases, antral follicles had no antral space in cross-section analysis, but were considered antral if they contained more than five granulosa cell layers. Follicles were defined as either healthy (intact germinal vesicle and nucleolus, oocyte with no more than three cytoplasmic vacuoles, intact basallamina) or atretic (apoptotic). If antral follicles contained at least twenty apoptotic granulosa cells (defined by the apoptotic bodies in the granulosa cell layer), disorganized granulosa cells, a fragmentation of the oocyte nucleus, or a degenerating oocyte, they were considered atretic [29].

### Western blot analysis

Mouse ovaries were homogenized in Radio-Immunoprecipitation Assay (RIPA) and Phenylmethanesulfonyl fluoride (PMSF) with a Teflon-glass homogenizer on ice. After centrifugation (12,000 rpm, 15 min at 4°C), the supernatants were collected for protein analysis. Protein concentrations were determined by the BCA Protein Assay Kit (Beyotime). The protein samples were separated by SDS–PAGE and transferred onto nitrocellulose membranes (BioTrace™ NT, USA). The membranes were blocked in 5% nonfat dry milk in Tris-Buffered-Saline with Tween 20 (TBST) for 1 hour and incubated with a primary antibody against SIRT1 (1:200 dilution), FOXO3a (1:200 dilution), SIRT6 (1:500 dilution), NRF1 (1:400 dilution), mTOR (1:200 dilution), phospho-mTOR (1:500 dilution), phospho-p70S6 kinase α (1:500 dilution), NFκB (1:600 dilution), p53 (1:600 dilution) or β-actin (1:5000 dilution) over-night at 4°C, followed by the incubation with a horseradish peroxidase-conjugated anti-rabbit or anti-mouse antibody (1:5000 dilution) at room temperature for 1 hour. Bands were visualized with a chemiluminescence reagent (Thermo Fisher Scientific, USA). Band intensities were analyzed using the Quantity One software (Bio-Rad Laboratories Pty. Ltd.). β - actin was used as a loading control.

### Statistical analysis

All results are expressed as the means ± S.E.M and analyzed by the SPSS 17.0 software. A one-way ANOVA was used to compare the data among groups. A *P*-value less than 0.05 was considered as statistical significance (*P* <0.05).

## Results

All mice were alive at the end of 24-week treatment, and no superficial abnormalities or tumors were found in the abdomen and other parts of the body.

### The overall status

The CHF mice displayed obese phenotype and showed unwieldy. In contrast, CR mice were thin and appeared increased physical activity; they were sensitive to food and foraged actively. Both the SRT and NAM mice had a similar body type to the CR mice after 6-week drug administration.

### Energy intake, body weight and visceral fat

**Figure 1:**
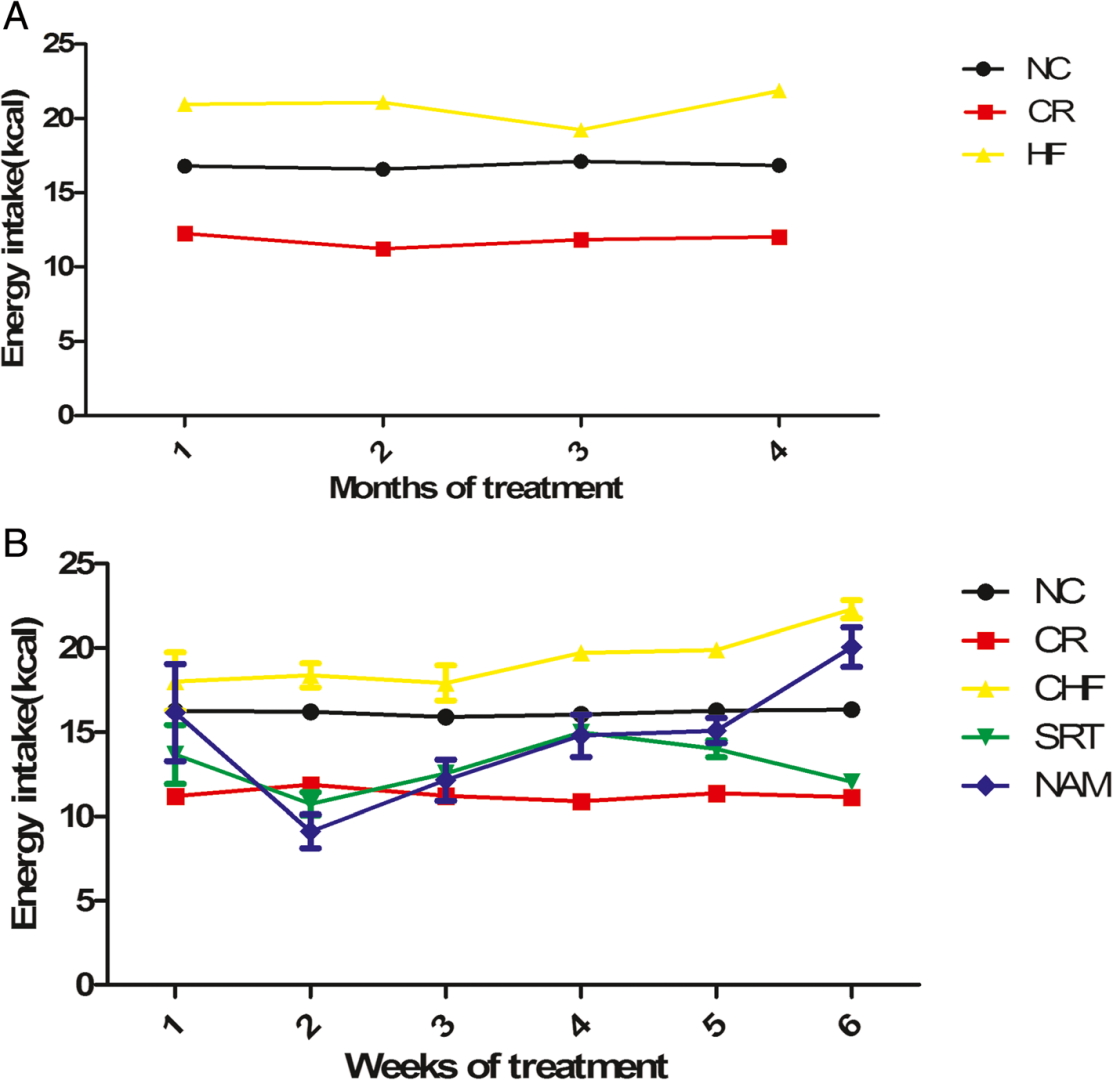
**Changes of energy intake among groups. (A)** Before drug administration. **(B)** During drug administration. Food intake was measured every day and the values were represented as means ± S.E.M.

The food intake of the NC mice remained constant throughout the course of the study, averaging 4.8 ± 0.02 g/d (standard chow, GE =16.8 ± 0.07 kcal). The intake of the CR group was controlled at an average of 3.4 ± 0.02 g/d (GE =11.8 ± 0.06 kcal). HF mice consumed 4.7 ± 0.04 g/d (HF diet, GE =20.8 ± 0.17 kcal) before drug administration (Figure 1A). The caloric consumption was higher in HF group than in the NC group (*P* < 0.001). During SRT1720 treatment, the energy intake of the SRT group gradually decreased in the first two weeks, and then increased in the middle two weeks. However, it decreased again and finally was similar to that of the CR group (12.1 ± 0.4 kcal/d vs. 11.1 ± 0.1 kcal/d, *P* > 0.05), lower than that of the NC group (16.4 ± 0.2 kcal/d, *P* < 0.001) (Figure 1B). The caloric intake of the NAM group decreased in the first two weeks, and then gradually increased in the following weeks. At the end of the treatment, it was close to that of the CHF group (20.1 ± 1.2 kcal/d vs. 22.6 ± 0.5 kcal/d, *P* > 0.05), but higher than that of the NC group (*P* < 0.001) (Figure 1B).

**Figure 2:**
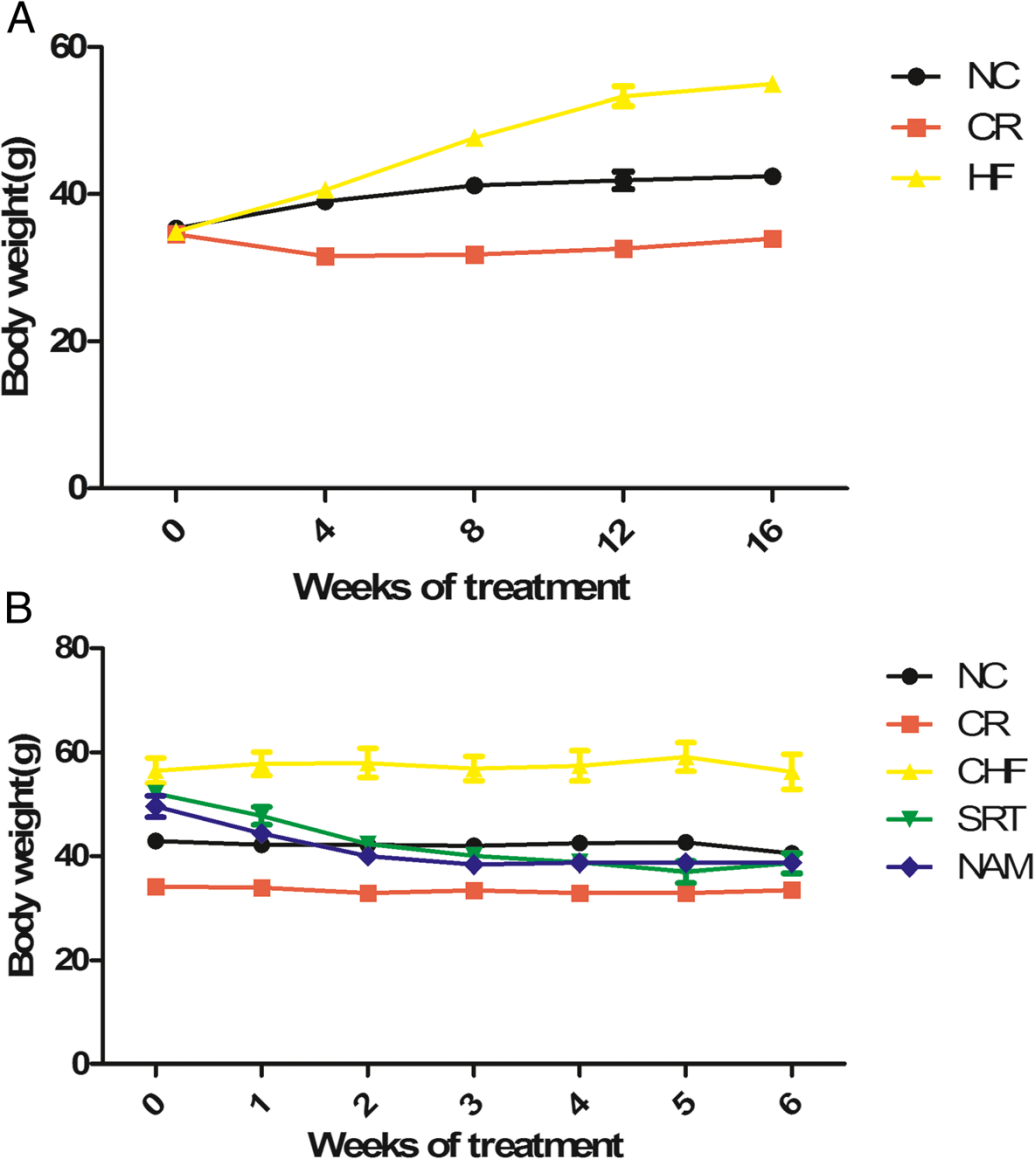
**Changes of body weight among groups. (A)** Before the drug administration. **(B)** During the drug administration. The values were represented as means ± SEM.

**Figure 3:**
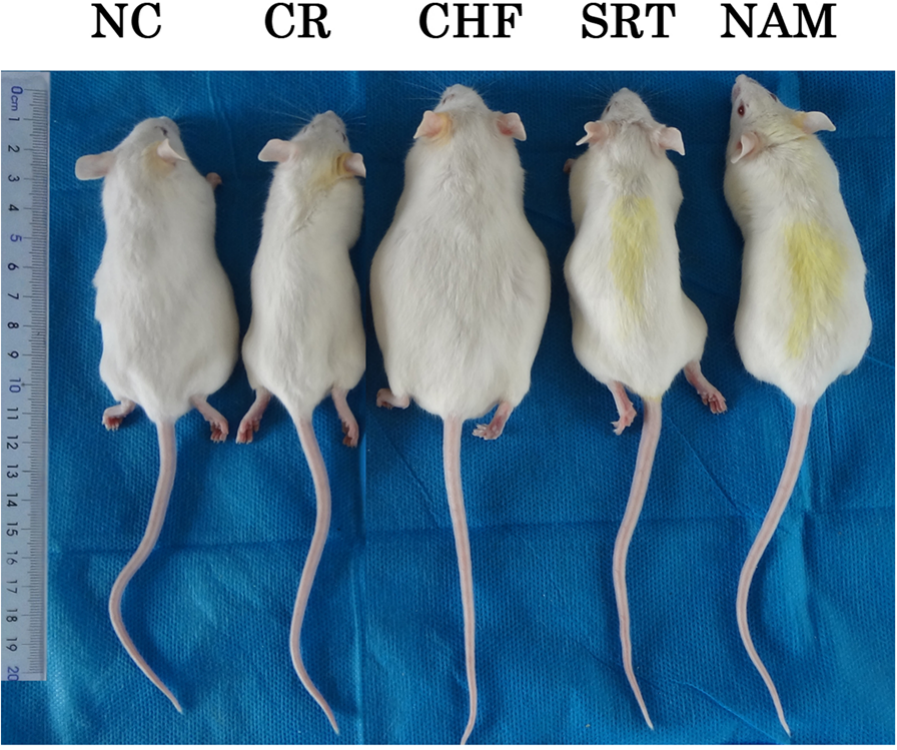
**Comparison of the shapes of mice between the groups at the end of the experiment.**

No difference was observed in the body weight of mice among the NC, CR and HF groups before the dietary treatment (*P* > 0.05). The body weight of the NC mice increased during the 26-week experiment (35.3 ± 0.5 g vs. 40.7 ± 1.7 g, *P* < 0.01), while that of the CR mice decreased slightly and remained relatively stable later (34.6 ± 0.7 g vs. 33.5 ± 1.6 g, *P* > 0.05). The body weight of the HF mice significantly increased from 34.9 ± 0.3 g to 55.0 ± 1.0 g during the four-month dietary treatment (*P* < 0.001) (Figure 2A) and they had 34% greater body weight than the NC mice, which were considered as moderate obesity [30,31]. Both the SRT group and the NAM group displayed a body weight dropped during the drug administration (38.7 ± 1.9 g vs. 52.1 ± 1.2 g, *P* < 0.05; 38.7 ± 1.5 g vs. 50.0 ± 2.1 g, *P* < 0.05), which were similar to the body weight of the CR group (*P* > 0.05) (Figure 2B). However, the body weight of the CHF group remained relatively stable (56.6 ± 2.1 g vs. 54.7 ± 3.4 g, *P* > 0.05) (Figure 3).

At the end of experiment, the perirenal fat pads were isolated and weighed as the visceral fat. The CHF mice had more visceral fat than the NC mice, while the visceral fat was less in the CR mice than in the NC mice. The SRT and NAM mice had similar visceral fat to the NC mice (Table 1).

**Table 1:** **Body weight, ovary weight and visceral fat in all groups at the end of treatment**

**Group**	**Body weight (g)**	**Ovary weight (mg)**	**Ovary/body weight (%)**	**Viscal fat (g)**
NC	40.7 ± 1.7	9.1 ± 0.8	0.022	0.25 ± 0.03
CR	33.5 ± 1.6*	5.9 ± 0.5*	0.018	0.11 ± 0.03*
CHF	56.3 ± 3.4***	18.7 ± 0.3***	0.032**	1.24 ± 0.20***
SRT	38.7 ± 1.9	5.9 ± 1.0*	0.015	0.32 ± 0.10
NAM	38.7 ± 1.5	11.7 ± 1.1	0.031*	0.33 ± 0.07

Data are means ± S.E.M. *P < 0.05, **P < 0.01, ***P < 0.001 compared with the NC group.

### The ovary weight

Both the CHF mice and the NAM mice had heavier ovaries and higher ratio of ovary weight to body weight than those of the NC mice. However, the gross ovary weight and ovary ratio of the SRT group were similar to those of the CR group, but less than those of the NC group (Table 1).

### Effect of SRT1720 and nicotinamide treatment on estrous cycles

The estrous cycles of all mice were examined before the treatment and only one of them represented an irregularly estrous cycle (>5 days). 100% (20/20) HF mice had exhibited a shortened estrous cycle or continuous estrus phase since the 8th week of diet treatment. However, after 6-week SRT1720 administration, 50% (3/6) SRT mice changed the continuous estrus phase to 3, 5 and 6 days, respectively. All the NAM and CHF mice maintained continuous estrus phase during the drug treatment. During the experiment, 87.5% (7/8) of the CR mice gradually exhibited an extended estrous cycle due to a prolonged diestrus phase and only one CR mouse kept a regular estrous cycle. Interestingly, two more CR mice and one SRT mice represented regularly again at the end of the study. Meanwhile, 75% (6/8) NC mice maintained regular estrous cycles until the end (Table 2).

**Table 2:** **Percentage of mice at different stages of the estrous cycle among groups**

**Age and estrous cycle stage**	**NC (n = 8)**	**CR (n = 8)**	**CHF (n = 8)**	**SRT (n = 6)**	**NAM (n = 6)**
	**%**	**%**	**%**	**%**	**%**
2 months					
Regular cycling	100	100	87.5	100	100
Irregular cycling	0	0	12.5	0	0
3 months					
Regular cycling	100	75	87.5	66.7	83.3
Irregular cycling	0	25	12.5	33.3	16.7
4 months					
Regular cycling	100	62.5	0	0	0
Irregular cycling	0	37.5	100	100	100
5 months					
Regular cycling	66.7	12.5	0	0	0
Irregular cycling	33.3	87.5	100	100	100
6 months					
Regular cycling	75	12.5	0	0	0
Irregular cycling	25	87.5	100	100	100
7 months					
Regular cycling	75	37.5	0	0	0
Irregular cycling	25	62.5	100	100	100
8 months					
Regular cycling	75	37.5	0	16.7	0
Irregular cycling	25	62.5	100	83.3	100

Note: The dietary treatment started at the beginning of 8 weeks (2 months) of age and stopped at the end of 32 weeks (8 months) of age. The drug administration started at the 26 weeks of age and lasted for 6 weeks.

### Effect of SRT1720 and nicotinamide treatment on ovarian follicular reserve

#### Comparison of the healthy follicles and atretic follicles among groups

**Figure 4:**
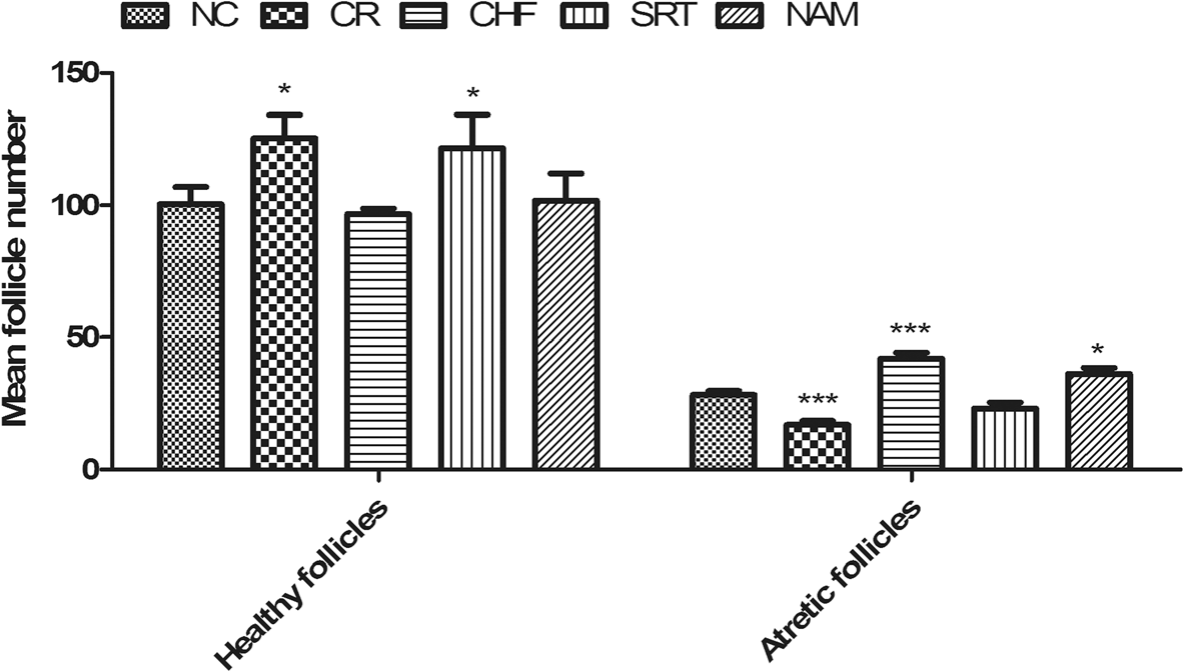
**Comparison of the healthy follicles and atretic follicles among groups.** Data were presented as mean ± S.E.M. *P < 0.05, **P < 0.01, ***P < 0.001 vs. the NC group.

HE staining results showed that mouse ovaries were mainly consisted of healthy follicles (primordial, primary, secondary and antral follicles), corpora lutea and atretic follicles. The number of healthy follicles in the SRT1720 group was similar to that of the CR group (121.7 ± 12.6 vs. 125.4 ± 8.7, *P* > 0.05), but more than that of the NC, CHF and NAM group (121.7 ± 12.6 vs. 100.4 ± 6.5, 96.6 ± 2.2 and 101.8 ± 10.3, *P* < 0.05, respectively). The number of atretic follicles in the SRT1720 group was similar to that of the NC group (23.2 ± 2.2 vs. 28.3 ± 1.6, *P* > 0.05), but less than that of the CHF group and the NAM group (23.2 ± 2.2 vs. 42.2 ± 2.4, 36.3 ± 2.3, *P* < 0.05), while the number of atretic follicles in the CR group was less than that of the NC group (16.9 ± 1.6 vs. 28.3 ± 1.6, *P* < 0.001) (Figure 4).

#### Comparison of the number of follicles at different stages among groups

**Figure 5:**
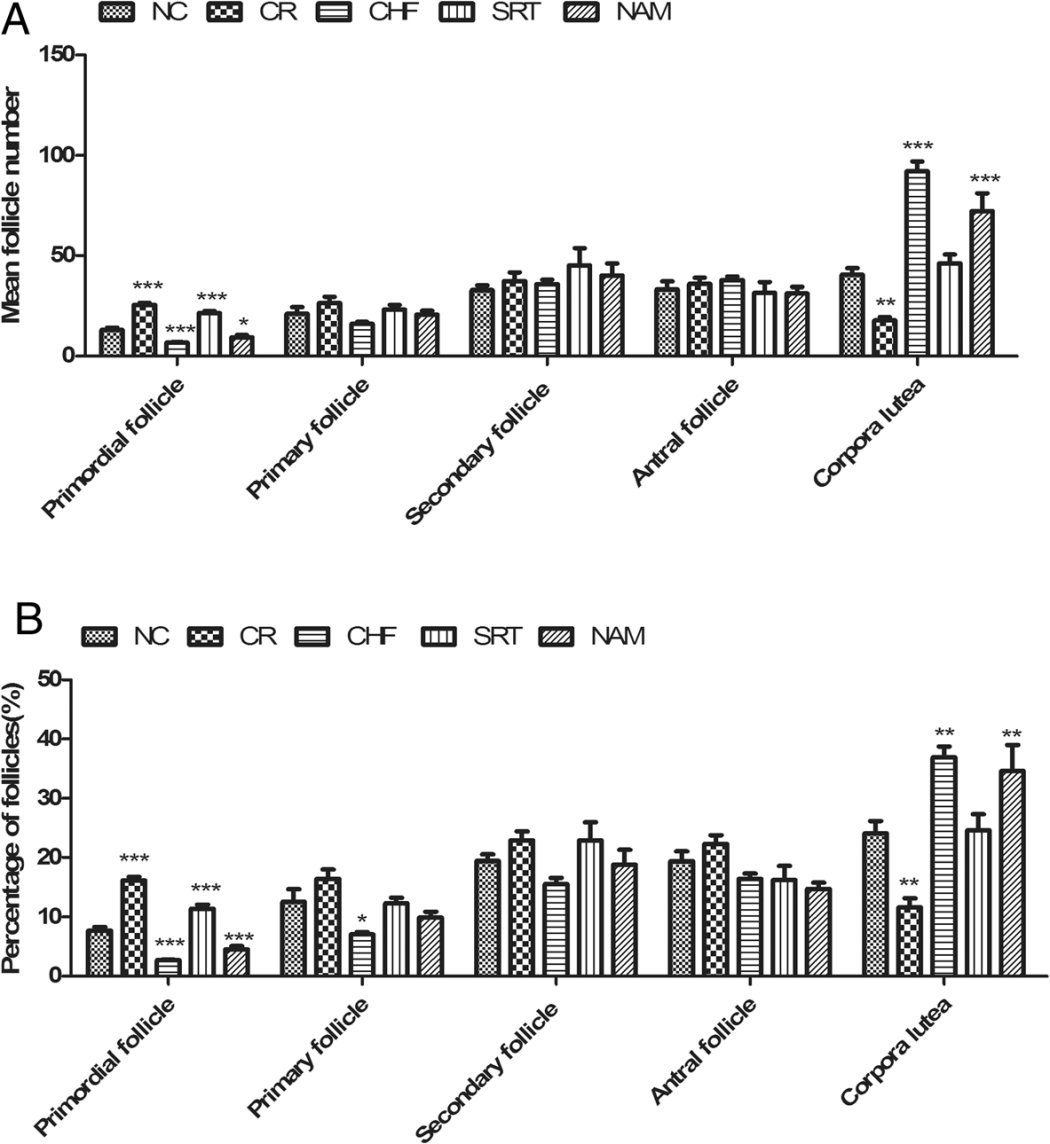
**Comparison of ovarian follicles at each stage among groups. (A)** The mean number of ovarian follicles at each stage among groups. **(B)** Percentage of ovarian follicles at each stage among groups. Data were presented as mean ± S.E.M. *P < 0.05, **P < 0.01, ***P < 0.001 vs. the NC group.

The mean number and percentage of primordial follicles in the SRT1720 group were more than those of the NC group (21.5 ± 1.1 vs. 13.1 ± 1.1; 11.4% ±0.7% vs. 7.7% ±0.6%, *P* < 0.001, respectively), while those of the CHF group and the NAM group were less than those of the NC group (6.8 ± 0.3, 9.5 ± 1.1 vs. 13.1 ± 1.1, *P* < 0.001, *P* < 0.05, respectively; 2.7% ±0.1%, 4.5% ± 0.6% vs. 7.7% ± 0.6%, *P* < 0.001, *P* < 0.01, respectively). Although the SRT1720 group had a similar mean number of primordial follicles to the CR group (21.5 ± 1.1 vs. 25.6 ± 1.0, *P* > 0.05), it had less percentage of primordial follicles than the CR group (11.4% ±0.7% vs. 16.2% ±0.6%, *P* < 0.001). The mean number and percentage of developing follicles (including primary, secondary and antral follicles) were comparable among groups (*P* > 0.05). The number and percentage of corpora lutea in the SRT1720 group were similar to those of the NC group (46.2 ± 4.3 vs. 40.5 ± 3.4; 24.6% ±2.8% vs. 24.1% ±2.1%, *P* > 0.05, respectively), but less than those of the CHF and NAM group (46.2 ± 4.3 vs. 92.1 ± 4.9, 72.3 ± 5.0, *P* < 0.001, respectively; 24.6% ±2.8% vs. 37.0% ±1.8%, 34.6% ±4.4%; *P* < 0.01, respectively). The CR group had less corpora lutea than the NC group (17.9 ± 4.7 vs. 40.5 ± 3.4; 11.6% ±1.5% vs. 24.6% ±2.8%, *P* < 0.01, respectively) (Figure 5).

### Western blotting analysis

**Figure 6:**
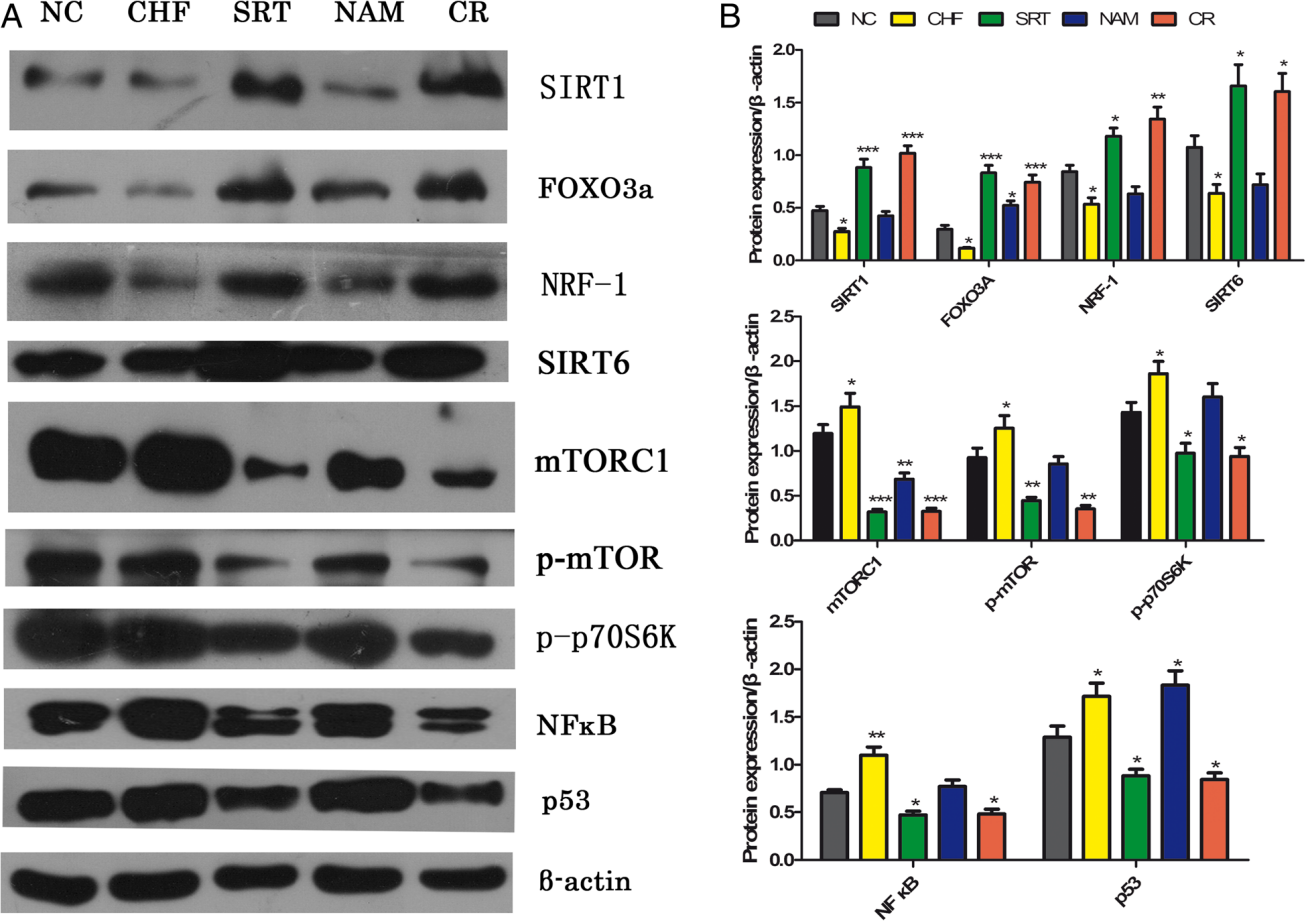
**The protein levels of SIRT1, FOXO3a, NRF-1, SIRT6, mTORC1, p-mTOR, p-p70S6K, NFκB and P53 in mouse ovaries by Western blot analysis. (A)** Western blot results. **(B)** Quantitative analysis of data in A. β-actin was used as internal control. Data are expressed as means ± S.E.M. *P < 0.05, **P < 0.01, ***P < 0.001 vs. the NC group.

To examine the activities of SIRT1/FOXO3a/NRF1-SIRT6, mTOR-p70S6K signaling, NFκB and p53 in the ovaries after SRT1720 and nicotinamide treatment, the protein expression of SIRT1, SIRT6, FOXO3a, NRF-1, mTORC1, p-mTOR, p-p70S6K, NFκB and p53 was measured by Western blotting. The result demonstrated that the level of SIRT1, SIRT6, FOXO3a and NRF-1 proteins significantly increased in the ovaries of the SRT and CR mice, whereas that of mTORC1, p-mTOR, p-p70S6K, NF-κB and p53 decreased compared to the NC mice. Contrarily, the CHF and NAM mice displayed a significant increase of mTORC1, p-mTOR, p-p70S6K, NFκB and p53, and a significant decrease of SIRT1, SIRT6, FOXO3a and NRF-1 proteins compared to the NC and SRT mice (Figure 6).

## Discussion

The epidemic of obesity is now recognized as one of the most important public health problems facing the world today and its impact on fertility is significant. As the prevalence of obesity is increasing, the number of women in the reproductive age who are becoming overweight and obese has the same trend. Obesity impacts at least 30% of reproductive aged women [32]. Weight-loss programs can improve fertility, hormones, ovulation in obese female [33]. CR is an effective way to lose weight and useful for prolonging the ovarian lifespan. Weight loss provides many benefits, but changing eating behavior and maintenance of ideal weight are difficult and hard to achieve [34,35]. Therefore, greater efforts are being devoted to understanding the mechanisms of CR-mediated regulation of ovarian follicle development so that it can provide new insight into extending ovarian lifespan and also into the potential therapeutic targets for obese females.

### High-fat diet induced obesity accelerated the ovarian follicle development and rate of follicle loss

In the present study, our data showed that obesity was effectively induced since adult (8 weeks old) in mice by *ad libitum* feeding of a high fat diet, for the CHF mice had greater body weight and visceral fat at the end of the study. Moreover, the CHF mice had less number and percentage of primordial follicles, and a greater number of corpora lutea and atretic follicles, suggesting that the high-fat diet induced obesity may accelerate the rate of follicle loss at least in three ways: i) stimulating the activation of primordial follicles; ii) promoting the development and maturity of ovarian follicles; iii) promoting follicle apoptosis. These results were coincident with our previous findings [10].

### SIRT 1 signaling was involved in the regulation of ovarian follicle development

Mammalian SIRT1, the ortholog of yeast Sir2, is a class III histone deacetylase (HDAC) whose activation is dependent on nicotinamide adenine dinucleotide in the nucleus [36,37]. It not only deacetylates histones, but also has a wide range of non-histone sustrates, such as the forkhead box class O (FOXO) family, p53 and nuclear factor κB (NFκB), etc. [38-40]. Accumulated evidence has revealed that SIRT1 is crucial for caloric restriction-induced longevity [41-43], and SIRT1 genetic variation is related to obesity [44], suggesting that SIRT1 is a key regulator of whole-body energy balance. SIRT1 also plays a role in reproductive biology. SIRT 1 transgenic mice showed phenotypes resembling CR and displayed prolonged lifespan, inhibited ovarian follicular development and delayed sexual maturity [45], whereas both male and female sirt1-null mice were barren [46-48]. FOXO3a is known as an important substrate of SIRT1. Mice with deletion of FOXO3a gene have been shown to have abnormal ovarian follicular development with early degeneration of oocytes, resulting in age-dependent infertility [49], whereas sexual maturity was delayed and follicle development was inhibited in oocyte specific FOXO3a transgenic mice [50]. Our previous study demonstrated that CR improved the follicle reserve and extended ovarian lifespan with increasing expression of SIRT1 and SIRT6 [51]. On the contrary, the level of SIRT1 and SIRT6 expression in the ovaries decreased in obese rats [10]. Kim et al. recently reported SIRT1 forms a complex with FOXO3a and NRF1 on the SIRT6 promoter to positively regulated expression of SIRT6 [52]. Our study also suggested that SIRT1/FOXO3a/NRF1- SIRT6 signaling may be involved in CR extending ovarian lifespan mechanisms [10].

Both SIRT 1 transgenosis and activators of SIRT 1 can mimic CR effect. However, it has remained elusive whether SIRT1 signaling plays a role in the development of ovarian follicles. Thus, we used SRT1720, the specific activator of SIRT1, to investigate its effect on the follicle development of the high-fat diet induced obesity mice. Our results showed that SRT1720 treatment caused an increase in the number and percentage of primordial follicles, which was comparable to CR treatment, suggesting that SRT1720 may inhibit the activation of primordial follicles like CR. Although the numbers of secondary and antral follicles were not significantly affected, the number and percentage of corpora lutea were decreased by the SRT1720 and CR treatment, suggesting that SRT1720 and CR may suppress follicle maturation. This may explain that the SRT1720-treated and CR ovaries were smaller than those of the control. Moreover, both the number and percentage of atretic follicles were significantly decreased by SRT1720, suggesting that SRT1720 may inhibit follicular atresia. Nicotinamide, a form of vitamin B3, is a product of Sir2-catalyzed deacetylation [53]. It has been clearly demonstrated that nicotinamide can inhibit Sir2 enzymes and down-regulate the expression of SIRT1 [54]. In the present study, the nicotinamide–treated mice had distinct features to the SRT or CR mice, their ovary weight, total number of follicles and mean number of follicles at different stages were comparable to that of the NC and CHF mice, suggesting that nicotinamide attenuated the effect of SRT1720. These results also suggest that SIRT1 signaling may play an important role in the mechanism of CR extending ovarian lifespan.

### SRT1720 treatment extended estrous cycle

It has been established that female reproductive aging is closely associated with a decreased ovarian follicle reserve and gradual loss in regular estrous cyclicity at middle age [55,56] Hence, we examined the status of estrous cycle in all groups. We found that the CR mice gradually displayed an extended estrous cycle due to a prolonged diestrus phase, while most HF mice exhibited a shortened estrous cycle or continuous estrus phase before drug treatment. After treated with SRT1720, 3 of the 6 SRT mice changed the continuous estrus phase to 3, 5 and 6 days, respectively. We supposed that the extended estrous cycle of the CR and SRT mice resulted from insufficient estrogen secreted by fewer mature follicles. This is in agreement with our follicle count results.

### SRT1720 treatment enhanced SIRT 1 signaling and attenuated mTOR signaling

mTOR (mammalian target of rapamycin,also known as Frap1 in mice and other mammals), a ubiquitous, evolutionarily conserved serine/threonine kinase, acts as a central regulator of eukaryotic growth and cell division in response to nutrient and growth factor cues. mTOR generates two distinct complexes: rapamycin sensitive mTOR complex 1 (mTORC1) and rapamycin insensitive mTORC2. Previous studies reported that mTORC1-S6K1-rpS6 signaling may be involved in the activation of mammalian primordial follicles [57-59] and was negatively regulated by SIRT1 [60].

With mammalian models of CR in our studies, we found that CR significantly enhanced the reserve of follicle pool by suppressing the activation of primordial follicles as well as decreased protein expression of mTOR and pS6K, suggesting that CR could inhibited mTOR-S6K signaling [10]. Interestingly, our results of the present study also showed that SRT1720 had similar effects with CR, in which SRT1720 not only enhanced the reserve of follicle pool, but also down-regulated mTOR signaling, suggesting that mTOR signaling may be negatively regulated by SIRT1 signaling. We found, moreover, in the present study that SRT1720 induced a decrease of energy intake by ~33.4% (about 66.6% energy intake of the CHF mice), meaning that the SRT1720-treated mice were in a CR condition. Consistently, the body weight of SRT1720-treated mice was significantly less than that of the CHF mice, although they ate the same food as the CHF mice. These data also suggest that the effect of CR is realized through the activation of SIRT1. Taking together, we speculate that SRT1720 may enhance the reserve of follicle pool by directly up-regulating SIRT1 signaling and thus down-regulating mTOR expression.

### SRT1720 treatment attenuated NFκB signaling

Physiological events within the ovary, including ovulation and corpus luteum formation and regression, have been described as controlled inflammatory events [61].It is now established that obesity causes a state of chronic low-grade inflammation. Compared to healthy lean individuals, overweight and obese individuals have higher pro-inflammatory cytokines, such as nuclear factor κB [62,63]. It may partly explain why the CHF mice had more corpus lutea and a higher expression of NFκB. NFκB is a downstream of SIRT1 and it activates several other pro-inflammatory cytokines (IL-6, TNF-α, IL-1β) [64]. A recent study reported that the specific SIRT1 activator SRT1720 exerted anti-inflammatory effects [65]. Consistently, our present study also found that SRT1720-treated mice, as well as the CR mice, displayed significantly decreased level of NFκB compared to the CHF mice, suggesting that SIRT1 may play an important role in the anti-inflammatory effect of CR and further contribute to ovarian follicle development.

### SRT1720 treatment inhibited p53 protein expression

P53, a tumor suppressor gene regulated by SIRT1-mediated deacetylation, is a positive regulator of apoptosis in its native form (wild-type). The expression of p53 protein in the apoptotic granulosa cells of atretic follicles suggests its possible role in atresia [66]. A study also showed that p53 played an important role in the regulation and selection of oocytes at checkpoints, such that oocytes that would otherwise be lost may persist when p53 was absent or reduced [67]. These data suggest that p53 may be associated with follicle atresia. SIRT1 regulates p53 acetylation and p53-dependent apoptosis [68]. Therefore, we examined the effect of CR and SRT1720 on p53 protein expression in the mouse ovary. The results showed that both CR and SRT1720 could inhibit p53 protein expression in the ovaries, which was probably due to the activation of SIRT1.

## Conclusions

Our present study suggests that SRT1720 treatment may promote the ovarian lifespan of HF diet-induced obesity female mice by suppressing the activation of primordial follicles, the follicle maturation and atresia via activating SIRT1 signaling and suppressing mTOR signaling. It may also reduce the inflammatory reaction via modulating NFκB signaling. We believe that a better understanding of the interrelationship between SIRT1 and mTOR signaling will promote the development of new pharmacological insights to treat metabolic diseases associated with obesity.

## Abbreviations

CR: Calorie restriction
CHF: Control high fat
FOXO3a: Forkhead box group O 3a
HF: High fat
H&E: Hematoxylin and eosin
mTOR: Mammalian target of rapamycin
mTORC: Mammalian target of rapamycin complex
NC: Normal control
NAM: Nicotinamide
NRF-1: Nuclear respiratory factor 1
NFκB: Nuclear factor κB
PAGE: Polyacry1 amide gelelectrophoresis
PBS: Phosphate buffer solution
p-p70S6K: Phosphor-p70 ribosomal protein S6 kinases
p-mTOR: Phosphor- mammalian target of rapamycin
PMSF: Phenylmethanesulfonyl fluoride
RIPA: Radio-immunoprecipitation assay
SIRT1: Silent information regulator 2 related enzyme 1
SIRT6: Silent information regulator 2 related enzyme 6
SDS: Sodium dodecyl sulfate
S.E.M: Standard error of mean
SPSS: Statistical package for the social sciences
TBST: Tris-buffered-saline with tween 20
